# miR-181a post-transcriptionally targets GRK2 to limit maladaptive signaling in cardiomyocytes

**DOI:** 10.3389/fcvm.2026.1821660

**Published:** 2026-04-22

**Authors:** Heidi Cho, Melissa Lieu, Erhe Gao, Stephanie M. Kereliuk, Samuel Slone, Thiele Osvaldt Rosales, Christina Liu, Maya Hoteit, Eric Barr, J. Kurt Chuprun, Walter J. Koch

**Affiliations:** 1 Department of Cardiovascular Sciences, Lewis Katz School of Medicine at Temple University, Philadelphia, PA, United States; 2 Department of Surgery, Duke University School of Medicine, Durham, NC, United States

**Keywords:** GRK2, miR-181a, microRNA, cardiomyocytes, cardiac remodeling, hypertrophy, oxidative stress, post-transcriptional regulation

## Abstract

**Background:**

Pathological upregulation of G protein-coupled receptor kinase 2 (GRK2) is a hallmark of heart failure and contributes to maladaptive signaling, hypertrophic remodeling, and cardiomyocyte death. MicroRNAs (miRNAs) are key post-transcriptional regulators of cardiac stress responses, however whether GRK2 is subject to miRNA targeting has not yet been fully established.

**Methods:**

miRNA microarray profiling was performed on mouse hearts two weeks after myocardial infarction. Bioinformatic target prediction analysis identified candidate miRNAs that are predicted to bind GRK2 in the 3′ untranslated region (UTR). Direct binding was assessed using luciferase reporter assays, and miR-181a was selected as the miRNA of interest to further pursue mechanistic and functional validation. miR-181a was overexpressed or inhibited in neonatal rat ventricular myocytes (NRVMs) and exposed to several modes of cellular stress to induce hypertrophy, hypoxia, and accumulation of reactive oxygen species. GRK2 expression, hypertrophic remodeling, oxidative stress, cell viability, and cyclic AMP (cAMP) signaling were assessed using quantitative PCR, immunoblotting, fluorescence imaging, and biochemical assays.

**Results:**

miR-181a directly targeted the GRK2 3′UTR and suppressed GRK2 expression at both mRNA and protein levels. miR-181a overexpression attenuated stress-induced hypertrophic gene expression, reduced cardiomyocyte cell size, decreased oxidative stress, improved survival under hypoxia, and enhanced cAMP production under *β*-AR stimulation. Conversely, inhibition of miR-181a resulted in sustained GRK2 expression, exacerbated hypertrophic signaling, and decreased cAMP production.

**Conclusion:**

These findings identify miR-181a as a functional post-transcriptional regulator of GRK2 that limits maladaptive signaling, hypertrophic remodeling, and cardiomyocyte injury. miR-181a-mediated GRK2 inhibition represents a potential therapeutic strategy for mitigating pathological signaling in heart failure.

## 1 Introduction

Significant remodeling of intracellular signaling networks that are essential for maintaining cardiac function under stress is a major hallmark of heart failure (HF). Following cardiac injury, *β*-adrenergic receptor (*β*-AR) signaling pathways become altered acutely due to heightened sympathetic nervous system (SNS) activation to improve contractile function. However, chronic activation of the SNS becomes progressively maladaptive over time. A key mediator of this transition is G protein-coupled receptor kinase 2 (GRK2), which becomes significantly upregulated in the failing myocardium (1–4). Elevated GRK2 promotes receptor desensitization, uncoupling, and downregulation, thereby diminishing *β*-AR responsiveness and contributing to the progressive decline in cardiac contractile function (2–4).

In addition to receptor desensitization, GRK2 has been shown to also have non-canonical roles in metabolic regulation, mitochondrial function, and cell survival pathways (5–7). As a result, increased level of GRK2 is not only a direct consequence of HF, but also a critical driver of disease progression. Extensive efforts have therefore focused on defining methods of therapeutic GRK2 inhibition, with genetic models, peptide inhibitors, and pharmacological strategies collectively demonstrating improved cardiac outcomes (3, 4, 8–11). Despite these advances, the endogenous mechanisms by which cardiomyocytes regulate GRK2 expression, particularly at the post-transcriptional level, remain incompletely understood.

One such potential mechanism involves post-transcriptional regulation by microRNAs (miRNAs). These small non-coding RNAs mediate gene expression by binding to complementary sequences within the 3′ untranslated regions (3′UTRs) of target mRNAs, leading to transcript degradation or translational repression (12–14). miRNAs are dynamically regulated in cardiovascular disease and influence multiple components of stress-induced signaling pathways, such as those of hypertrophy and cell survival (15–17). In the context of *β*-AR signaling involving GRK2, endogenous miRNA-mediated inhibition could serve as an important compensatory mechanism to attenuate excessive GRK2 upregulation and preserve signaling homeostasis. However, this layer of regulation remains poorly defined.

In this study, we investigated whether specific miRNAs regulate GRK2 in cardiomyocytes and whether modulation of these miRNAs influences downstream signaling and cellular stress responses. Using miRNA microarray analysis of mouse hearts two weeks post-myocardial infarction (MI), combined with target prediction approaches, we identified miR-181a-5p as a candidate miRNA with potential relevance to GRK2 regulation. We therefore sought to test our hypothesis that miR-181a directly represses GRK2 expression and modulates cardiomyocyte responses to stress by limiting maladaptive cardiomyocyte stress responses.

## 2 Materials and methods

### 2.1 Experimental animals

All animal procedures were carried out according to National Institute of Health Guide for the Care and Use of Laboratory Animals and approved by the Animal Care and Use Committee of Temple University. Adult male C57BL/6J (9–12 weeks) mice purchased from Jackson Laboratory were utilized for all *in vivo* experiments. Mice were euthanized by isoflurane inhalation and cervical dislocation, as approved by the American Veterinary Medical Association (AVMA).

### 2.2 Echocardiography

Cardiac function was measured at baseline and 2 weeks post-myocardial infarction (MI). Echocardiography was performed using the Vevo 2100 imaging system from VisualSonics. Mice were anesthetized with 3% (v/v) isoflurane inhalation and a MS400 transducer was used for assessment of cardiac function. Mid-ventricular short-axis images were acquired for M-mode imaging.

### 2.3 Myocardial infarction

MI surgery was performed as previously described (18). Briefly, mice were anesthetized with 2% isoflurane inhalation using an isoflurane vaporizer (Viking Medical) without intubation. While on a heated surface, a small incision was made on the left side of the chest to expose the fourth intercostal space. The heart was gently pushed out of the thoracic cavity, and a slipknot was tied around the left coronary artery (LCA) using a 6-0 silk suture. The heart was placed back with manual evacuation of the pneumothoraxes. The skin was closed by suture. Sham operated animals did not receive LCA occlusion. The mouse was then removed from isoflurane inhalation and closely monitored for recovery.

### 2.4 miRNA microarray analysis

Male C57BL/6J mice aged 9–12 weeks underwent sham or MI surgery. At 2 weeks post-MI, total RNA was extracted from the left ventricular (LV) tissue of the mouse myocardium. miRNA expression profiling was performed by LCSciences.

### 2.5 Luciferase reporter assay

Ad293 cells were transfected with 300 ng of pmiRGLO-GRK2-3′UTR or pmiRGLO-mutGRK2-3′UTR plasmids using Lipofectamine 2000 (Thermo Fisher Scientific). Constructs were generated using the pmiRGLO Dual-Luciferase miRNA Target Expression Vector (Promega) containing either the full-length murine GRK2 3′UTR or a mutant 3′UTR sequence inserted via XbaI/SalI cloning and sequence-verified (GenScript). Following 48 h culture, stable integration was selected using G418 (1.0–1.4 mg/mL), individual resistant clones were expanded and screened, and one stable clone for each construct (Ad293-GRK2 and Ad293-mutGRK2) was established. Cell lines were maintained in 1.0 mg/mL G418, which was omitted during assays.

For luciferase experiments, Ad293-GRK2 or Ad293-mutGRK2 cells were plated at 1 × 10^5^ cells/well in 24-well plates, serum-starved (1% FBS) for 5 h, and transfected with 25 pmol miRNA mimic or inhibitor using Lipofectamine RNAiMAX (Thermo Fisher Scientific) in Opti-MEM. After 48 h, cells were lysed in Luciferase Lysis Buffer (25 mM Tris-phosphate, 4 mM EGTA, 1% Triton X-100, 10% glycerol, 2 mM DTT). Protein content was measured by Bradford assay (Bio-Rad), and equal protein amounts were assayed in triplicate using the Dual-Glo Luciferase Assay System (Promega). Firefly and Renilla luciferase activities were recorded on an Infinite M1000 PRO plate reader (Tecan), and values were normalized to untransfected cell background controls.

### 2.6 Neonatal rat ventricular myocyte (NRVM) isolation

Primary NRVMs were isolated from 1 to 2 day old Sprague Dawley rat pups (*n* = 20) as previously described (19). Briefly, ventricles were excised, minced, and subjected to three serial digestions in ADS buffer containing pancreatin and collagenase II at 37 °C, shaking at 80 rpm. Supernatants from the second and third digestions were combined with Ham's F-10 containing 10% FBS, and centrifuged. Cells were resuspended in Ham's F-10 complete medium, filtered through a 70 μm strainer, and pre-plated for 2 h to reduce fibroblast contamination. Non-adherent cardiomyocytes were collected, counted via trypan blue exclusion, and seeded onto Corning primaria culture plates. Cells were maintained in Ham's F-10 complete medium (10% horse serum, 5% FBS, 1% penicillin-streptomycin) on Day 1, followed by Ham's F-10 with 5% FBS, 1% penicillin-streptomycin medium on Day 2 onward, and treated as described for each experiment.

### 2.7 miRNA mimic and inhibitor transfection

Synthetic miRNA mimics and inhibitors for miR-181a-5p, miR-181b-5p, miR-181c-5p, miR-181d-5p and Kaposi's sarcoma–associated herpesvirus miR-K12-3-5p (miR-K12, Qiagen) were used to overexpress or inhibit miRNAs. A scrambled, non-targeting control was used for miRNA mimic experiments (Scr mimic, Thermo Fisher Scientific, 4464058). For miRNA inhibitor experiments, a scrambled inhibitor negative control was used (Scr inh, Thermo Fisher Scientific, 4464076). Transfections were performed with Lipofectamine RNAiMAX (Thermo Fisher Scientific) according to the manufacturer's protocol. NRVMs were transfected with the miR-181a mimic (Thermo Fisher Scientific, MC10421), miR-181b mimic (Thermo Fisher Scientific, MC12442), miR-181c mimic (Thermo Fisher Scientific, MC10181), miR-181d mimic (Thermo Fisher Scientific, MC12522) miR-K12 mimic (Thermo Fisher Scientific, MC12583), or anti-miR-181a inhibitor (Thermo Fisher Scientific MH10421) in Opti-MEM I reduced serum medium (Thermo Fisher Scientific) for 24–48 h before subsequent treatments. Control wells received RNAiMAX with nuclease-free water only.

### 2.8 Phenylephrine (PE) treatment

To induce hypertrophic signaling, NRVMs were treated with 10 µM phenylephrine (PE; Santa Cruz sc-203677) for 48 h following 24 h of miRNA transfection. Vehicle (H_2_O)-treated cells served as controls. Following treatment, cells were harvested for RNA and protein analysis or prepared for immunofluorescence imaging.

### 2.9 Wheat germ agglutinin (WGA) staining and cell size quantification

Cardiomyocyte hypertrophy was assessed by measuring cell surface area following plasma membrane labeling with fluorescently conjugated wheat germ agglutinin (WGA). NRVMs were seeded in a 24-well plate at 2 × 10^5^ cells per well and treated with PE as described above. Live cells were incubated with Alexa Fluor 594-conjugated WGA (5 *μ*g/mL; Thermo Fisher Scientific) and Hoechst 33342 nuclear stain (1 *μ*g/mL; Thermo Fisher Scientific) diluted in phosphate-buffered saline (PBS) for 15 min at 37 °C, protected from light. Cells were washed three times with PBS and subsequently fixed in 4% paraformaldehyde (Thermo Fisher Scientific) for 15 min at room temperature, followed by three additional PBS washes. Fluorescence images were acquired using an EVOS M7000 Imaging System (Thermo Fisher Scientific) with equal exposure settings across conditions. Cardiomyocyte surface area was quantified using ImageJ (NIH) by tracing cell boundaries from WGA-stained membranes. For each experimental condition, two technical replicates were analyzed per biological replicate, with six non-overlapping fields of view imaged per technical replicate. Experiments were performed using three independent biological replicates, defined as separate NRVM isolations. Cell area measurements were averaged within each technical replicate and subsequently averaged within each biological replicate prior to statistical analysis.

### 2.10 Hypoxia treatment

To simulate ischemic stress, NRVMs were transfected with miRNA mimics or inhibitors for 48 h and then placed in a modular hypoxia chamber (1% O_2_, 5% CO_2_, 94% N_2_; Billups-Rothenberg) for 16 h. Control cells were maintained under normoxic conditions. After treatment, cells were collected for RNA and protein extraction or subjected to viability assays.

### 2.11 Cell viability assay

Cell viability was assessed using the LIVE/DEAD Viability/Cytotoxicity Assay Kit (Thermo Fisher Scientific) according to the manufacturer's instructions. Following hypoxia exposure, cells were incubated with 2 µM calcein-AM (green fluorescence, viable cells) and 4 µM SYTOX Deep Red (deep red fluorescence, dead cells) for 30 min at room temperature. Images were captured using the EVOS M7000 (Thermo Fisher Scientific) fluorescence microscope and analyzed with ImageJ (NIH). The percentage of viable cells was calculated as the ratio of calcein-positive to total (calcein+SYTOX) cells. Approximately 2,000 total cells were counted per condition for each biological replicate.

### 2.12 Measurement of intracellular reactive oxygen species (ROS)

NRVMs were transfected with miR-181a mimic or scrambled control oligonucleotides using Lipofectamine RNAiMAX (Thermo Fisher Scientific) for 48 h. Following transfection, oxidative stress was induced by treatment with 10 *μ*M of chelerythrine chloride (Santa Cruz sc-3547) for 30 min. After treatment, cells were incubated with CellROX Deep Red reagent (Invitrogen) diluted in pre-warmed culture medium at a final concentration of 5 μM and incubated at 37 °C for 30 min protected from light. Nuclei were co-stained with Hoescht 33342 (1 *μ*g/mL, Invitrogen). Cells were then washed with PBS and imaged immediately in live-cell conditions.

Fluorescence images were acquired using the EVOS M7000 imaging system (Thermo Fisher Scientific) with identical exposure settings across all experimental conditions. Mean fluorescence intensity (MFI) was quantified using ImageJ software (NIH). For each condition, six fields of view were analyzed per biological replicate, and background fluorescence was subtracted prior to analysis. Data were averaged within each biological replicate prior to statistical analysis.

### 2.13 Western blot analysis

Cells were lysed in RIPA buffer supplemented with protease and phosphatase inhibitor cocktails. Protein concentrations were measured using the Pierce BCA Protein Assay Kit. Equal amounts of protein were separated by SDS-PAGE and transferred to nitrocellulose membranes. Membranes were stained with Revert 700 Total Protein Stain (LI-COR) and imaged using an Odyssey DLx Imaging System (LI-COR). The total protein stain was removed with Revert Destaining Solution (LI-COR) then washed with Revert 700 Wash Solution (LI-COR). The membranes were subsequently blocked in TBS Intercept Blocking Buffer (LI-COR) for 1 h before incubating overnight at 4 °C with primary antibodies for GRK2 (Santa Cruz Biotechnology Cat# sc-13143, RRID:AB_626751, 1:1,000), phospho-Akt (Cell Signaling Technology Cat# 4060, RRID:AB_2315049, 1:1,000), or pan Akt (Cell Signaling Technology Cat# 2920, RRID:AB_1147620, 1:1,000). Membranes were incubated with secondary antibodies conjugated to fluorescent dyes IRDye 680 or IRDye 800 (LI-COR, 1:10,000) for 1 h at room temperature. Protein bands were visualized using an Odyssey DLx Imaging System (LI-COR).

### 2.14 Quantitative real-time PCR (qPCR)

Total RNA extraction was performed using the RNeasy Mini Kit (Qiagen) according to the manufacturer's protocol. For mRNA analysis, cDNA was synthesized using the iScript cDNA Synthesis Kit (Bio-Rad) and quantitative PCR was performed using iQ SYBR Green Supermix (Bio-Rad). For miRNA detection, cDNA was generated using the miRCURY LNA RT Kit (Qiagen), and quantitative PCR was performed using the miRCURY LNA SYBR Green PCR Kit (Qiagen). Both analyses were performed on a QuantStudio 3 Real-Time PCR System (Thermo Fisher Scientific). Relative gene expression was calculated using the 2^-*ΔΔ*Ct method. Expression of target genes for mRNA was normalized to 18S rRNA and miRNA expression was normalized to 5S rRNA. Primer sequences used for mRNA and miRNA quantification are listed in Tables 1, 2.

**Table 1:** Primer sequences used for mRNA analysis by quantitative PCR.

Target	Species	Forward primer (5′-3′)	Reverse primer (5′-3′)
GRK2	Rat	CTGCCAGTGGAAGAATGTAGAGC	ACCCATAGACCTCACCGAAGC
Nppa	Rat	AGGAGAAGATGCCGGTAG	GCTTTTCAAGAGGGCAGA
Nppb	Rat	TATCTCAAGCTGCTTTGGG	TACAACAACTTCAGTGCGT
Myh7	Rat	TCATCACCAGAATCCAGGC	AATCAGGGAGTCTCTG
PTEN	Rat	TGAGTTCCCTCAGCCATTGCCT	GAGGTTTCCTCTGGTCCTGGTA
18s rRNA	Rat	TCAAGAACGAAAGTCGGAGG	GGACATCTAAGGGCATCAC

**Table 2:** Primer mixes for miRNA PCR assay.

miRNA	Assay ID (Qiagen)
miR-181a-5p	YP00206081
Kshv-miR-K12-3-5p	YP00205823
5S rRNA	YP00203906

### 2.15 Cyclic adenosine monophosphate (cAMP) assay

Intracellular cAMP levels were measured using the Direct cAMP ELISA Kit (Enzo Life Sciences). NRVMs were pretreated with 1 mM 3-isobutyl-1-methylxanthine (IBMX; Sigma-Aldrich I7018) for 10 min to inhibit phosphodiesterases, followed by stimulation with increasing doses of isoproterenol (0, 10^−7^, 10^−6^, 10^−5^, 10^−4^ M; Sigma-Aldrich I6504) for 5 min. Cells were lysed and cAMP concentrations were determined according to the manufacturer's instructions. Data were normalized to total protein content determined by BCA assay (Thermo Fisher Scientific).

### 2.16 Statistical analysis

All experiments were performed in at least three independent biological replicates unless otherwise stated. Data are expressed as mean ± SEM. Statistical analyses were performed using GraphPad Prism 10. Comparisons between two groups were made using unpaired two-tailed t-tests. Multiple comparisons were analyzed using one-way or two-way ANOVA with the appropriate *post hoc* tests. Statistical significance was defined as *p* < 0.05.

## 3 Results

### 3.1 Identification of candidate miRNAs predicted to regulate GRK2 expression

**Figure 1:**
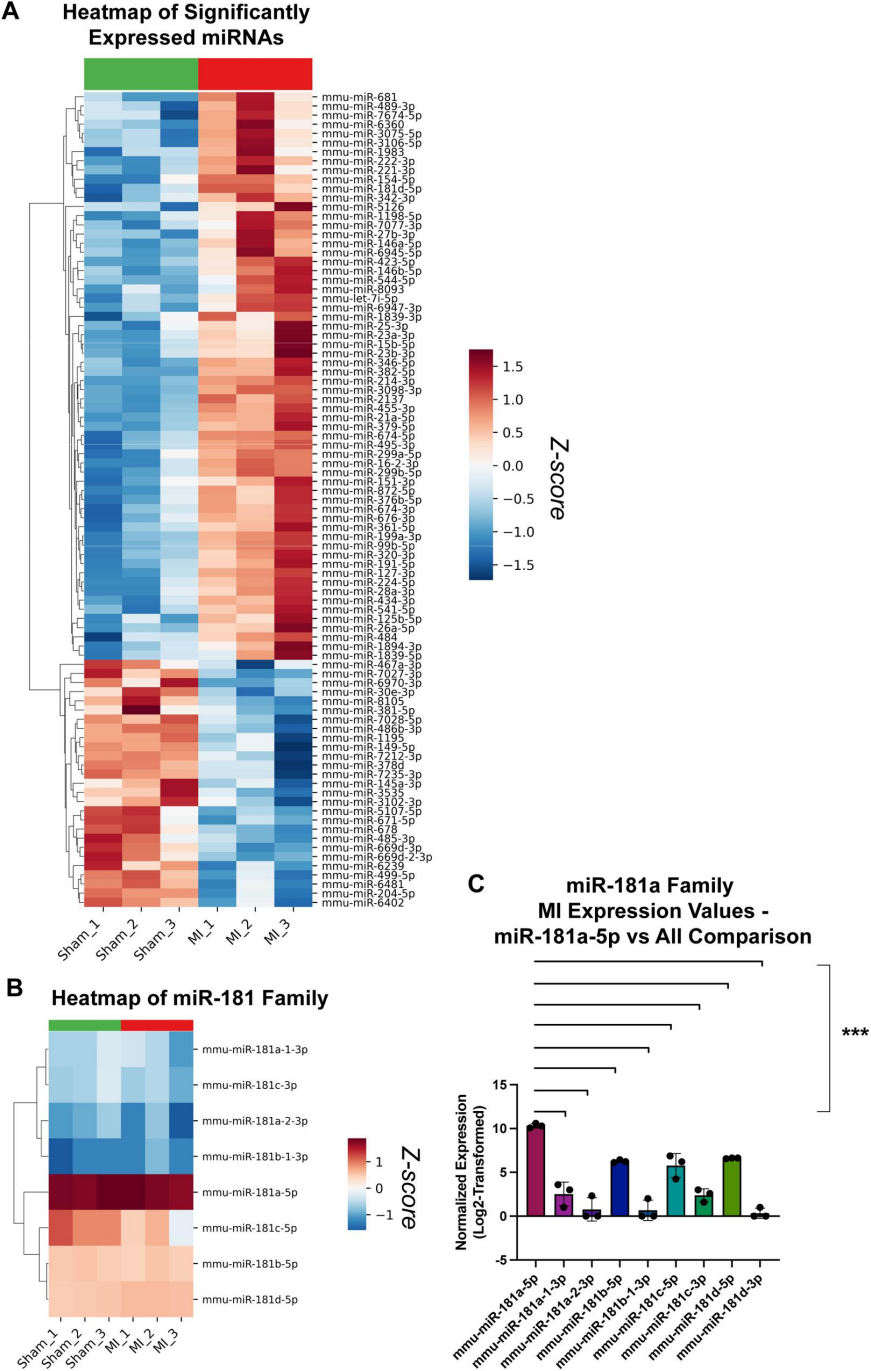
miRNA microarray screening identifies miR-181a as a highly expressed candidate regulator of GRK2. **(A)** Clustered heatmap of significantly expressed miRNAs identified by miRNA microarray analysis of LV tissue harvested from sham-operated and MI mice (*n* = 3–5 per group). miRNAs displayed met the significance threshold (*p* < 0.05) as determined by one-way ANOVA. **(B)** Clustered heatmap displaying the normalized expression values of the identified miR-181 family transcripts from the miRNA microarray analysis of sham and MI samples. **(C)** One-way ANOVA comparing normalized expression of miR-181-5p relative to other miR-181 family transcripts identified from the microarray analysis (*** *p* < 0.001). Heatmap analyses was performed on log2-transformed and z-score normalized values from the miRNA microarray analysis.

To identify whether miRNAs contribute to GRK2 regulation following cardiac injury, we performed miRNA microarray profiling on LV tissue harvested from mice two weeks after MI or sham surgery. Cardiac dysfunction following MI was first confirmed by echocardiography, which demonstrated a marked reduction in ejection fraction (EF) relative to sham-operated controls (Supplementary Figure S1). As expected, MI mice exhibited variable degrees of systolic dysfunction. To minimize biological variability, three MI mice with comparable levels of cardiac impairment (EFs of 24.39%, 26.57%, and 24.59%) were selected for RNA isolation and subsequent miRNA microarray analysis. Differential expression analysis revealed a broad pattern of miRNA remodeling in MI hearts, with several transcripts significantly up- or downregulated relative to sham controls (Figure 1A).

Because GRK2 expression is increased after MI (10, 20), we next sought to identify miRNAs with predicted binding sites within the GRK2 3′UTR. TargetScan (v8.0) analysis identified several candidate miRNAs with conserved seed matches, ranked by context++ score percentile (Table 3). Among these, members of the miR-181 family ranked highly based on context++ score percentiles (>80), including miR-181a-5p (miR-181a), which ranked in the 82nd percentile, indicating strong predicted targeting efficacy.

**Table 3:** Predicted miRNAs that target the GRK2 3′UTR identified by targetScan analysis. .

miRNA	Position in the UTR	Seed match	Context++ score	Context++ score percentile	Weighted context++ score	Conserved branch length
mmu-miR-181c-5p	1,020–1,026	7mer-m8	−0.14	85	−0.13	3.229
mmu-miR-181d-5p	1,020–1,026	7mer-m8	−0.13	82	−0.12	3.229
mmu-miR-181b-5p	1,020–1,026	7mer-m8	−0.13	82	−0.12	3.229
mmu-miR-181a-5p	1,020–1,026	7mer-m8	−0.13	82	−0.12	3.229

Candidate miRNAs predicted to bind the GRK2 3′UTR were identified using the TargetScan 8.0 database. miRNAs are ranked by context++ score percentile, with higher percentiles indicating stronger predicted targeting efficacy based on seed match type, evolutionary conservation, and surrounding sequence features. Only miRNAs with a context++ score percentile greater than 80 were included.

We then examined expression patterns of all detectable miR-181 family members within the microarray dataset. Hierarchical clustering of normalized expression values (Figure 1B) demonstrated that although individual family members exhibited distinct expression profiles, miR-181a was consistently the most abundantly expressed transcript in both sham and MI hearts. Quantitative comparison across family members confirmed that miR-181a expression was significantly higher than other miR-181 transcripts (Figure 1C). Notably, miR-181a expression was not significantly altered following MI, suggesting that its selection was based on its high basal abundance and strong predicted targeting of GRK2 rather than differential expression alone.

### 3.2 miR-181a directly binds the GRK2 3′UTR

**Figure 2:**
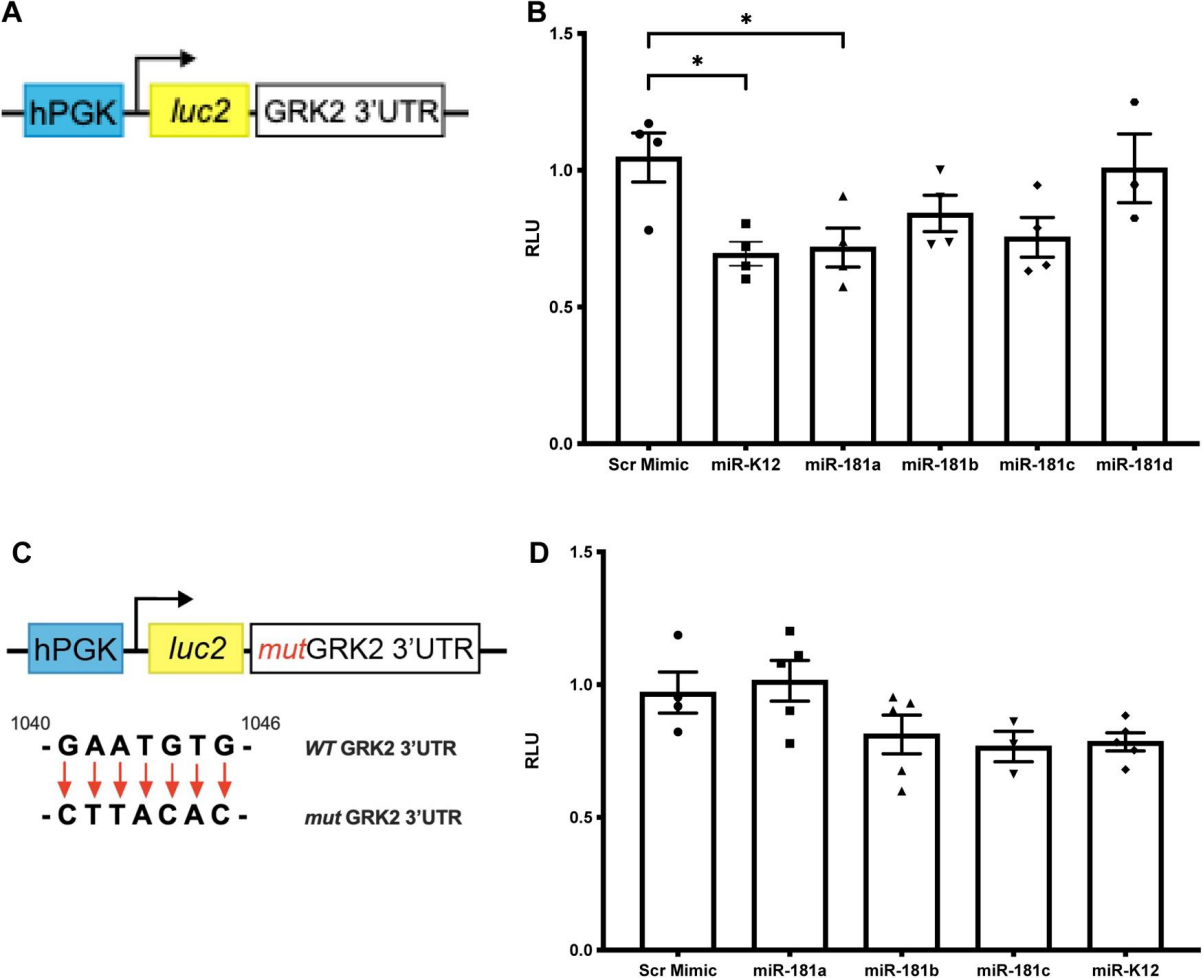
miR-181a directly targets the GRK2 3′UTR. Luciferase reporter assays were performed to evaluate direct miRNA-mediated regulation of the GRK2 3′UTR. **(A)** Schematic of the luciferase reporter construct containing the full-length GRK2 3′UTR downstream of the luciferase gene. **(B)** Luminescence measurements in Ad293 cells co-transfected with the GRK2 3′UTR luciferase reporter and either scrambled mimic or indicated miRNA mimics. miR-181a significantly reduced luciferase activity compared with scrambled control, whereas miR-181b, miR-181c, and miR-181d did not significantly alter luminescence. miR-K12, a previously validated GRK2-targeting miRNA, significantly reduced luciferase activity and was included as a positive control in this initial screen. **(C)** Schematic of the luciferase reporter construct containing a mutated GRK2 3′UTR lacking the predicted miRNA seed binding site. **(D)** Luminescence measurements in cells transfected with the mutant GRK2 3′UTR luciferase reporter and scrambled mimic or indicated miRNA mimics. Mutation of the GRK2 3′UTR abolished miRNA-mediated repression, as miR-181a, miR-181b, miR-181c, and miR-K12 no longer reduced luciferase activity compared with scrambled controls. miR-181d was not included in mutant construct experiments due to the absence of significant repression in the wild-type GRK2 3′UTR assay. Data are presented as individual biological replicates with mean ± SEM. *n* = 3 independent experiments, each performed in technical duplicate. Statistical significance was determined using one-way ANOVA followed by Dunnett's multiple comparisons test comparing each miRNA to the scrambled control, where * *p* < 0.05.

Bioinformatic prediction and expression profiling provide valuable guidance in selecting miRNA candidates, however these analyses alone cannot establish regulation by direct binding. Thus, we evaluated whether candidate miRNAs directly bind the GRK2 3′UTR using luciferase reporter assays. Ad293 cells stably expressing a luciferase construct containing the full-length GRK2 3′UTR (Figure 2A) were transfected with individual miRNA mimics predicted to target GRK2 with high context++ score percentiles (>80). Among the miR-181 family members, miR-181a significantly reduced luciferase activity compared to scrambled control, whereas miR-181b, miR-181c, and miR-181d did not significantly decrease reporter activity (Figure 2B). As expected, miR-K12, a previously validated GRK2-targeting miRNA (21), robustly suppressed luciferase activity and was included as a positive control in this initial screen (Figure 2B).

To confirm that miR-181a-mediated repression occurs through direct binding to the GRK2 3′UTR, parallel experiments were performed in Ad293 cells stably expressing a luciferase reporter containing a mutated GRK2 3′UTR lacking the predicted miRNA seed binding site (Figure 2C). Because miR-181 family members share a conserved seed sequence, miR-181b and miR-181c were included in the mutant reporter assay despite only modest effects in the wildtype (WT) construct, to exclude low affinity or context-dependent interactions. miR-181d was not advanced to mutant testing because it did not reduce luciferase activity in the WT GRK2 3′UTR reporter.

Consistent with seed sequence-dependent targeting, mutation of the GRK2 3′UTR eliminated miRNA-mediated repression. In contrast to the WT reporter, miR-181a did not reduce luciferase activity in the mutant construct and instead exhibited a slight, non-significant increase relative to scrambled control (Figure 2D). Similarly, miR-181b, miR-181c, and the positive control miR-K12 produced small reductions in luciferase activity that did not reach statistical significance (Figure 2D). These findings indicate that miR-181a-mediated repression of GRK2 requires an intact 3′UTR binding site, supporting direct and sequence-specific targeting. Together, these results establish miR-181a as a functional regulator of GRK2 and provide a mechanistic basis for subsequent studies examining its role in cardiomyocyte stress responses.

### 3.3 Overexpression of miR-181a modulates GRK2 expression and AKT activation

**Figure 3:**
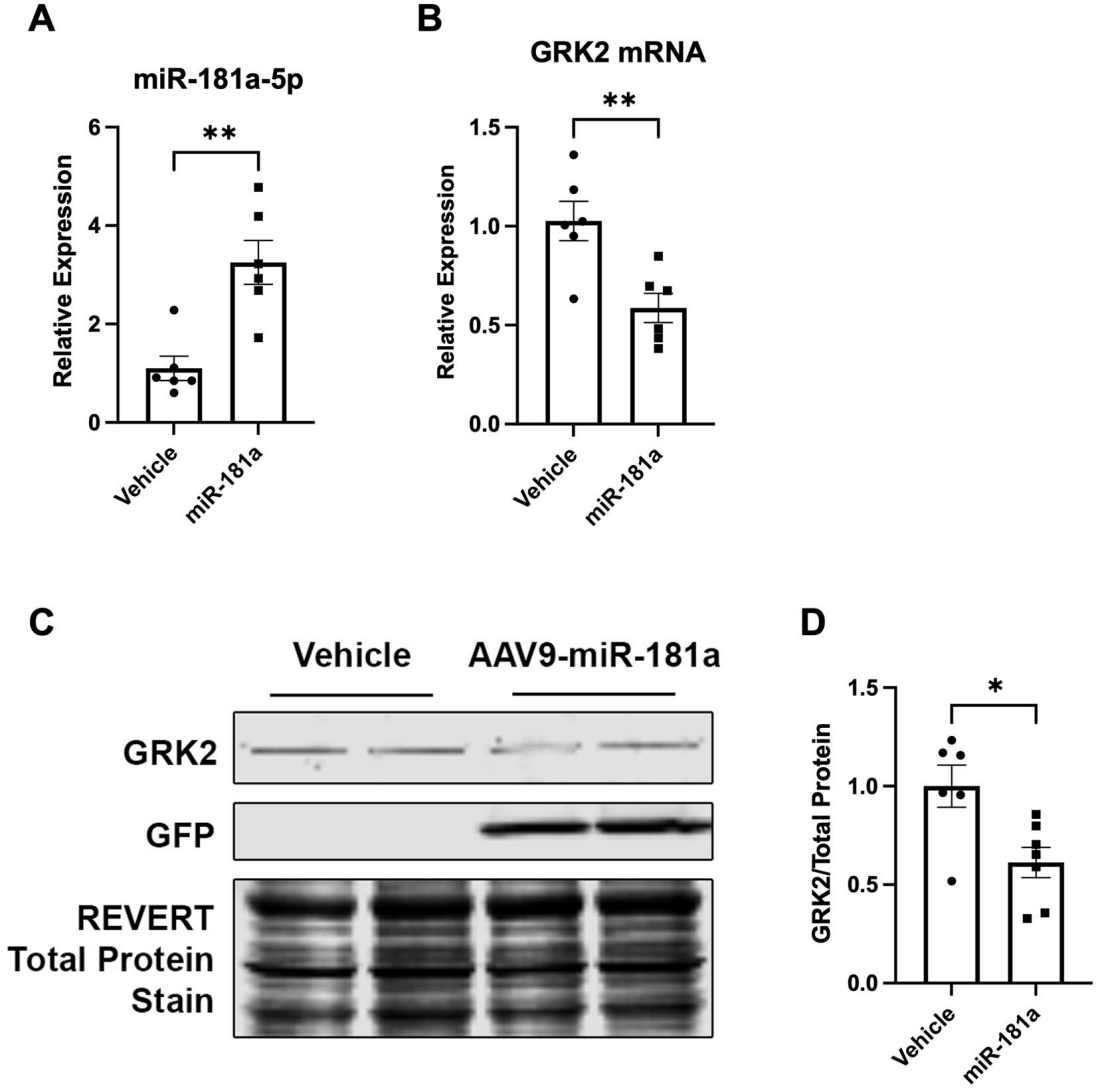
Cardiac AAV9-mediated miR-181a overexpression reduces GRK2 expression *in vivo*. Wild-type mice were injected retro-orbitally with AAV9-cTNT-miR-181a-CMV-GFP or vehicle (PBS). Hearts were harvested two weeks post-injection. **(A)** RT-qPCR analysis confirming increased miR-181a-5p expression in cardiac tissue following AAV9 delivery. **(B)** GRK2 mRNA levels measured by RT-qPCR demonstrating reduced expression in miR-181a-injected hearts compared to controls. **(C)** Representative immunoblot showing GFP expression and GRK2 protein levels in cardiac lysates. **(D)** Quantification of GRK2 protein normalized to total protein, demonstrating decreased GRK2 abundance following miR-181a overexpression. Data are presented as mean ± SEM. *n* = 6 mice per group. Statistical analysis was performed using unpaired t-test, where * *p* < 0.05 and ** *p* < 0.01.

To establish whether miR-181a regulates GRK2 expression in the adult heart *in vivo*, wild-type mice were administered AAV9-cTNT-miR-181a via retro-orbital injection. Two weeks post-delivery, cardiac miR-181a expression was significantly increased compared to controls (Figure 3A), confirming successful transduction. Consistent with our *in vitro* findings, GRK2 mRNA (Figure 3B) and protein levels (Figures 3C, D) were significantly reduced in miR-181a-overexpressing hearts. These data demonstrate that miR-181a suppresses GRK2 expression *in vivo* and support the physiological relevance of our subsequent mechanistic studies in cardiomyocytes.

**Figure 4:**
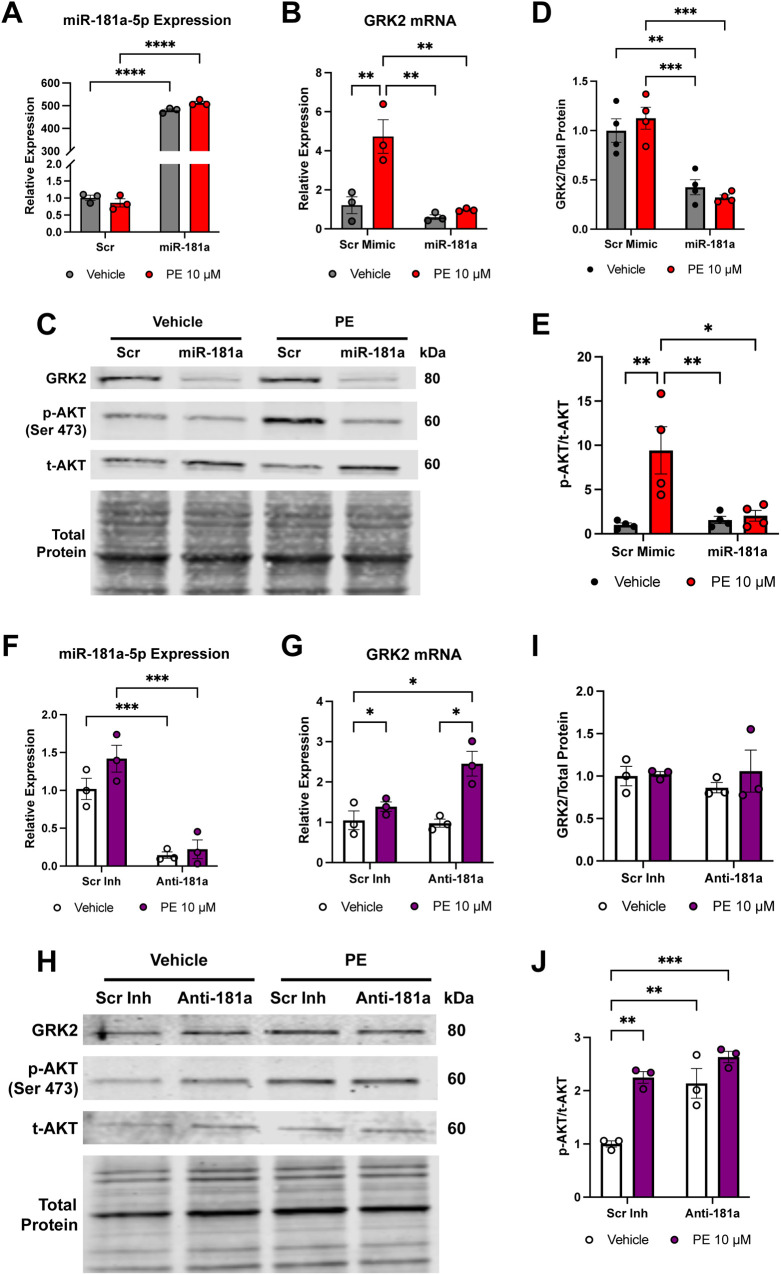
miR-181a suppresses GRK2 expression and modulates AKT signaling in cardiomyocytes subjected to PE stimulation. Neonatal rat ventricular myocytes (NRVMs) were transfected with miR-181a mimic, anti-miR-181a, or their respective scrambled controls for 24 h and subsequently treated with phenylephrine (PE; 10 µM) for 48 h to induce hypertrophic stress. **(A)** RT-qPCR confirmation of miR-181a-5p overexpression following mimic transfection. **(B)** GRK2 mRNA expression measured by RT-qPCR demonstrating PE-induced upregulation in scrambled control cells, which was significantly attenuated by miR-181a overexpression. **(C)** Representative immunoblot showing GRK2, p-AKT, and t-AKT protein levels in scrambled control and miR-181a-overexpressing NRVMs under basal and PE-stimulated conditions. **(D)** Quantification of GRK2 protein normalized to total protein following miR-181a overexpression under basal and PE-stimulated conditions. **(E)** Quantification of phosphorylated AKT (Ser473) relative to total AKT following miR-181a overexpression under basal and PE-stimulated conditions. **(F)** RT-qPCR confirmation of miR-181a inhibition following anti-miR-181a transfection. **(G)** GRK2 mRNA expression following miR-181a inhibition. **(H)** Representative immunoblot showing GRK2, p-AKT, and t-AKT protein levels following miR-181a inhibition under basal and PE-stimulated conditions. **(I)** Quantification of GRK2 protein normalized to total protein following miR-181a inhibition under basal and PE-stimulated conditions. **(J)** Quantification of phosphorylated AKT (Ser473) relative to total AKT following miR-181a inhibition under basal and PE-stimulated conditions. Data are presented as mean ± SEM (*n* = 3 independent NRVM isolations). Statistical analysis was performed using two-way ANOVA with Tukey's *post hoc* multiple comparisons test, where * *p* < 0.05; ** *p* < 0.01; *** *p* < 0.001; **** *p* < 0.0001.

Next, we sought to evaluate whether the direct targeting of the GRK2 3′UTR by miR-181a results in functional regulation of GRK2 signaling in response to hypertrophic stress. NRVMs were transfected with either miR-181a mimic or scrambled control and treated with phenylephrine (PE, 10 *μ*M, 48 h). Efficient overexpression of miR-181a following mimic transfection was confirmed by qPCR (Figure 4A). As an independent validation of miR-181a overexpression or inhibition, we assessed the mRNA levels of a previously established miR-181a target phosphatase and tensin homolog (PTEN) (22). Overexpression of miR-181a was associated with significantly reduced PTEN mRNA levels, whereas inhibition of miR-181a was associated with increased PTEN levels, confirming effective functional modulation of miR-181a (Supplementary Figures S2A, B).

Following PE stimulation, GRK2 mRNA levels were significantly increased compared to the scrambled mimic control (Figure 4B), consistent with a previous report that also demonstrated increased levels of GRK2 following PE treatment (23). This upregulation of GRK2 mRNA was significantly attenuated with miR-181a overexpression (Figure 4B). Although GRK2 protein levels did not increase with PE stimulation, overexpression of miR-181a robustly decreased GRK2 protein under both vehicle and PE-stimulated conditions (Figures 4C, D). Because GRK2 is known to negatively regulate AKT signaling (24), we also assessed AKT phosphorylation. PE treatment increased p-AKT/total AKT ratios in scrambled control cells, whereas miR-181a overexpression blunted this response (Figure 4E).

To complement these findings, we performed parallel experiments using a miR-181a inhibitor. As expected, inhibition of endogenous miR-181a decreased miR-181a levels (Figure 4F) and resulted in the opposite phenotype of miR-181a overexpression. Inhibiting miR-181a led to elevated GRK2 mRNA expression following PE stimulation (Figure 4G). Consistent with miR-181a overexpression, PE stimulation did not increase GRK2 protein levels, however inhibition of miR-181a resulted in sustained GRK2 protein levels, consistent with vehicle-treated controls (Figures 4H, I). Further, inhibition of miR-181a exacerbated PE-induced AKT phosphorylation (Figure 4J), supporting a physiological role for endogenous miR-181a in limiting GRK2 expression and restricting maladaptive signaling.

Together, these results demonstrate that miR-181a is both necessary and sufficient to suppress GRK2 expression and modulate AKT signaling in cardiomyocytes exposed to pro-hypertrophic stimulation.

### 3.4 PE-induced cardiomyocyte hypertrophy is attenuated by miR-181a

Because GRK2 and AKT signaling pathways contribute to pathological cardiomyocyte hypertrophy (23), we next evaluated the impact of miR-181a on hypertrophic gene expression and cell size in response to PE stimulation. NRVMs were transfected with miR-181a mimic or inhibitor and treated with PE. Expression levels of hypertrophic markers were determined by qPCR, and cellular hypertrophy was evaluated by WGA staining to quantify cell surface area.

**Figure 5:**
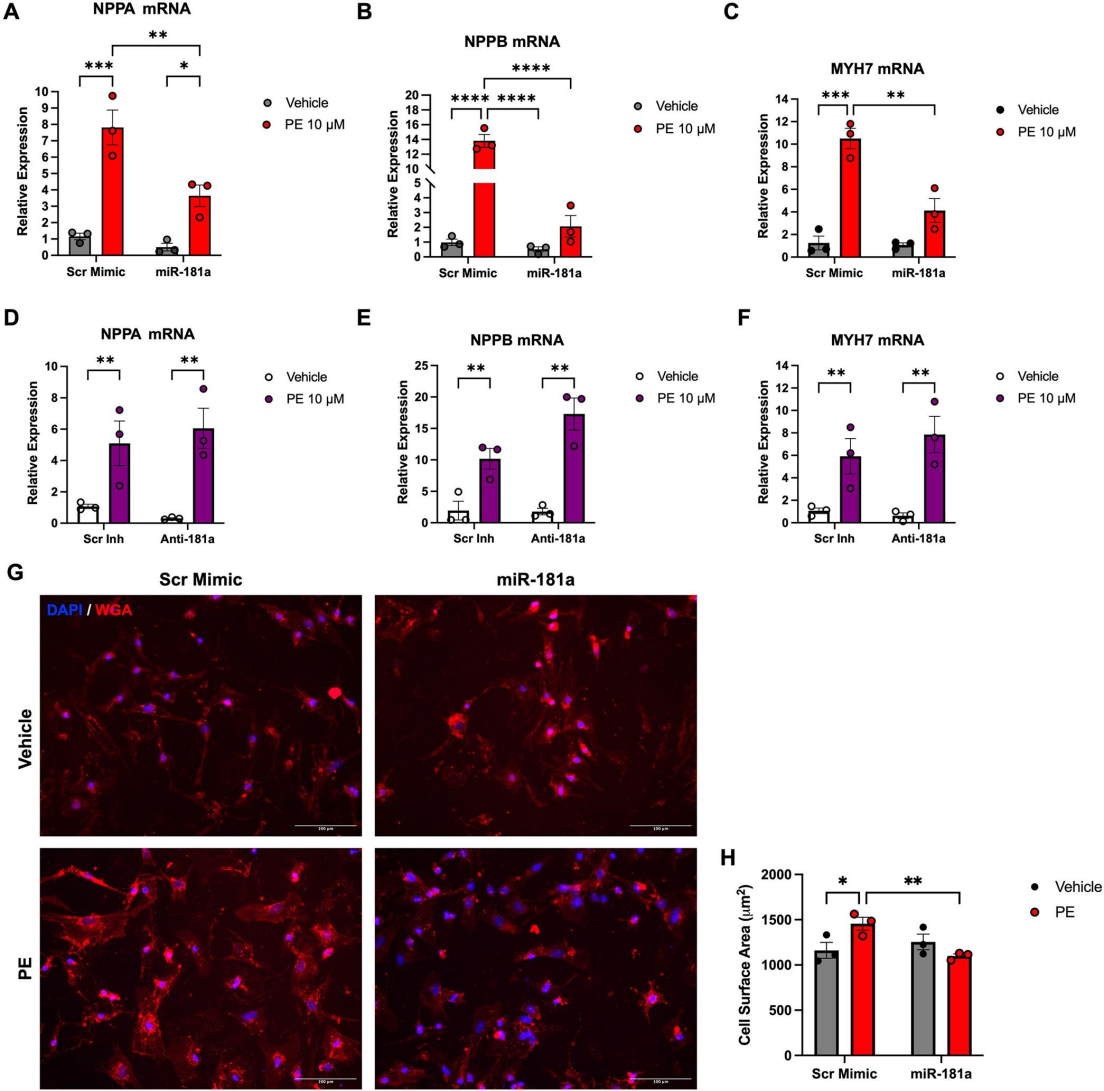
miR-181a attenuates PE-induced hypertrophic gene expression in cardiomyocytes. NRVMs were transfected with miR-181a mimic, anti-miR-181a, or their respective scrambled controls for 24 h and subsequently treated with 10 µM of PE for 48 h to induce hypertrophic stress. **(A–C)** RT-qPCR analysis showing PE-induced increases in NPPA **(A)**, NPPB **(B)**, and MYH7 **(C)**, which were attenuated by miR-181a overexpression. **(D–F)** Inhibition of miR-181a exacerbated PE-induced increases in NPPA **(D)**, NPPB **(E)**, and MYH7 **(F)** expression compared with scrambled inhibitor controls. **(G)** Representative WGA-stained images of cardiomyocytes. **(H)** Quantification of cardiomyocyte surface area. Data are presented as individual biological replicates with mean ± SEM. *n* = 3 independent NRVM isolations. For WGA analysis, two technical replicates per condition were analyzed per biological replicate, with six non-overlapping fields of view imaged per technical replicate; cell area measurements were averaged within technical replicates and then averaged per biological replicate. Statistical analysis was performed using two-way ANOVA with Tukey's *post hoc* multiple comparisons test, where * *p* < 0.05; ** *p* < 0.01; *** *p* < 0.001; **** *p* < 0.0001.

Consistent with a hypertrophic response, PE stimulation significantly increased mRNA levels of atrial natriuretic peptide (NPPA), brain natriuretic peptide (NPPB), and *β*-myosin heavy chain (MYH7) in scrambled control cells (Figures 5A–C). Overexpression of miR-181a significantly attenuated PE-induced upregulation of all three hypertrophic markers, indicating suppression of the hypertrophic transcriptional program. In contrast, inhibition of endogenous miR-181a produced the opposite effect. Transfection with anti-miR-181a further exacerbated PE-induced increases in NPPA, NPPB, and MYH7 expression compared with scrambled inhibitor controls (Figures 5D–F).

Morphological assessment supported these transcriptional findings. WGA staining revealed that PE stimulation significantly increased cardiomyocyte surface area in scrambled mimic controls, whereas miR-181a overexpression significantly attenuated PE-induced increases in cell size, consistent with suppression of hypertrophic remodeling (Figures 5G, H). In contrast, inhibition of miR-181a did not reduce cardiomyocyte surface area following PE stimulation (Supplementary Figures S3A, B). These findings indicate that miR-181a acts as a negative regulator of cardiomyocyte hypertrophic signaling, with overexpression conferring protection against both molecular and morphological features of PE-induced hypertrophic remodeling, whereas inhibition of miR-181a fails to rescue pathological increases in cell size.

### 3.5 Cardiomyocyte survival is enhanced by miR-181a following exposure to hypoxia

**Figure 6:**
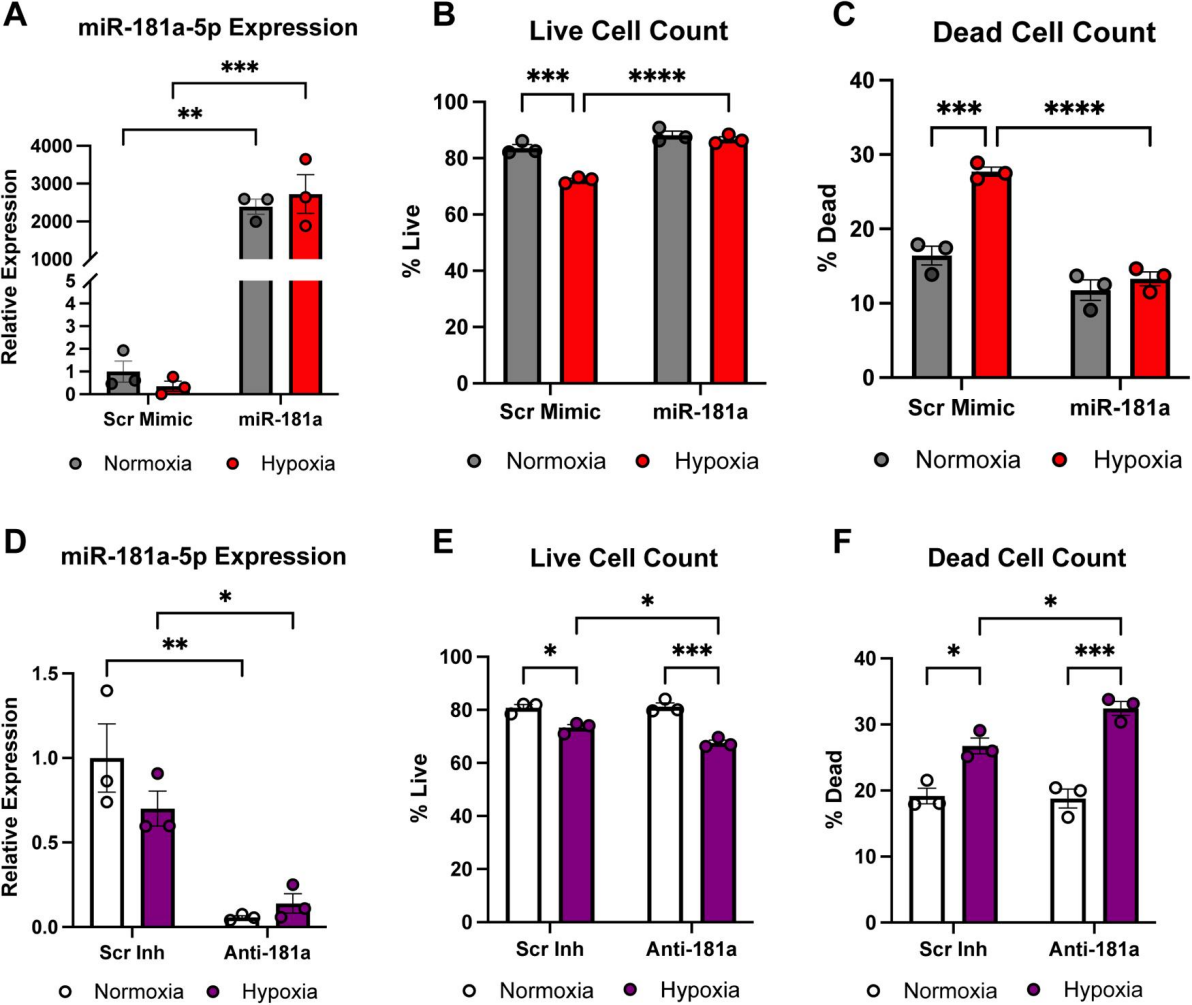
miR-181a enhances cardiomyocyte survival under hypoxic stress. NRVMs were transfected with miR-181a mimic or inhibitor and exposed to normoxia or hypoxia. **(A)** RT-qPCR confirmation of robust overexpression of miR-181a under both normoxic and hypoxic conditions. **(B)** Live/Dead viability analysis demonstrated a significant reduction in viable cells following hypoxia in scrambled control cells, whereas miR-181a overexpression preserved cell viability, with no difference between normoxia and hypoxia. **(C)** Hypoxia significantly increased dead cell number in scrambled controls; miR-181a overexpression significantly reduced cell death under both basal and hypoxic conditions. NRVMs were transfected with scrambled anti-miR or anti-miR-181a. **(D)** RT-qPCR confirmed effective inhibition of endogenous miR-181a. **(E, F)** miR-181a inhibition resulted in the opposite phenotype of overexpression, with exaggerated hypoxia-induced reductions in viable cells and increased dead cell number compared with scrambled inhibitor controls. For Live/Dead assays, *n* = 3 biological replicates, with 6 fields per well and 3 wells per condition per experiment analyzed. qPCR values represent *n* = 3 biological replicates, each run in technical triplicate. Data are presented as mean ± SEM (*n*=3 biological replicates). Statistical analysis was performed using two-way ANOVA with Tukey's *post hoc* test, where * *p* < 0.05; ** *p* < 0.01; *** *p* < 0.001; **** *p* < 0.0001.

Because hypertrophic remodeling is often accompanied by increased susceptibility to ischemic stress, we next examined whether miR-181a also influences cardiomyocyte survival under hypoxic conditions. NRVMs were transfected with either miR-181a mimic or scrambled control and subjected to overnight hypoxia. Robust overexpression of miR-181a following mimic transfection was confirmed by qPCR in both normoxic and hypoxic conditions (Figure 6A).

Exposure to hypoxia significantly reduced cell viability in the scrambled control group, consistent with hypoxia-induced cell death (Figure 6B, Supplementary Figure S4A). In contrast, miR-181a overexpression preserved cardiomyocyte viability, as live cell counts remained unchanged between normoxia and hypoxia in the miR-181a group. Correspondingly, hypoxia induced a significant increase in dead cell number in scrambled controls, whereas miR-181a overexpression significantly reduced cell death under both normoxic and hypoxic conditions (Figure 6C, Supplementary Figure S4A), indicating enhanced cellular resilience.

To assess whether endogenous miR-181a is required for hypoxic tolerance, complementary loss-of-function experiments were performed. Inhibition of miR-181a was verified by qPCR (Figure 6D). Suppression of endogenous miR-181a resulted in decreased cell viability after hypoxia (Figure 6E, Supplementary Figure S4B) and a significant increase in dead cell number (Figure 6F, Supplementary Figure S4B), demonstrating that cardiomyocytes are more susceptible to hypoxic injury following inhibition of miR-181a. These results indicate that miR-181a promotes cardiomyocyte survival under hypoxic stress, supporting a protective role for miR-181a in conditions relevant to ischemic heart disease.

### 3.6 Oxidative stress-induced ROS are reduced by miR-181a

Since oxidative stress is a key driver of cardiomyocyte injury in HF (25), we next examined whether miR-181a limits ROS accumulation in response to chelerythrine-induced oxidative stress. Chelerythrine, a benzophenanthridine alkaloid and protein kinase C inhibitor, has been shown to induce oxidative stress in cardiomyocytes, in part through mitochondrial dysfunction and increased ROS production (26). NRVMs were transfected with miR-181a mimic or scrambled control and subsequently treated with chelerythrine to induce oxidative stress. Intracellular ROS levels were assessed using CellROX staining followed by fluorescence imaging and quantification.

**Figure 7:**
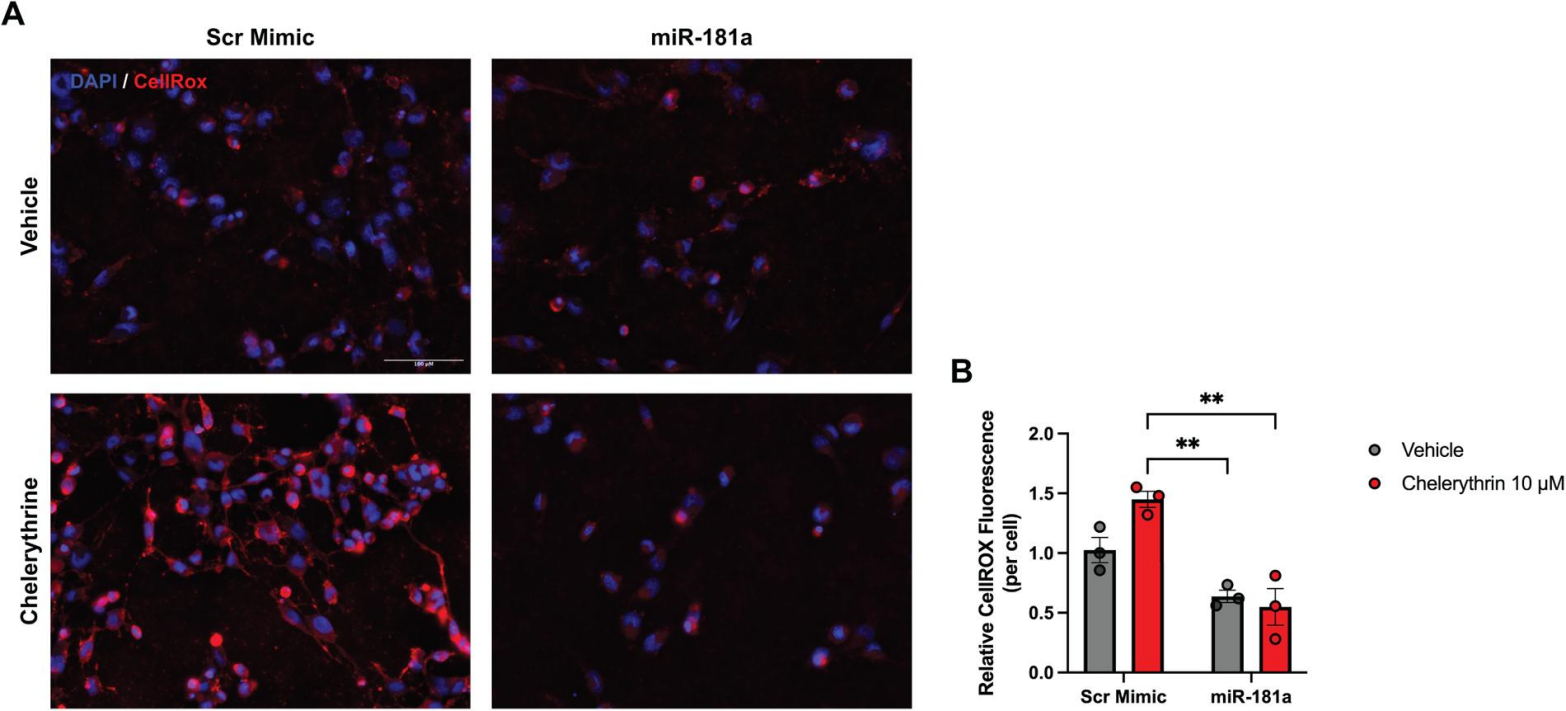
miR-181a attenuates oxidative stress in cardiomyocytes. NRVMs were transfected with miR-181a mimic or scrambled control for 48 h and subsequently treated with chelerythrine to induce oxidative stress. Intracellular reactive oxygen species (ROS) levels were assessed using CellROX staining. **(A)** Representative fluorescence images of CellROX staining following chelerythrine treatment. Increased fluorescence intensity indicates elevated oxidative stress. **(B)** Quantification of mean fluorescence intensity (MFI) demonstrating a significant increase in oxidative stress in scrambled control cells after chelerythrine treatment, which was significantly attenuated by miR-181a overexpression. Data are presented as individual data points with mean ± SEM. *n* = 3 independent biological replicates. Statistical significance was determined two-way ANOVA with Tukey's *post hoc* test, where ** *p* < 0.01; *** *p* < 0.001.

Quantitative analysis of mean fluorescence intensity (MFI) confirmed a significant increase in oxidative stress in chelerythrine-treated scrambled control cells, whereas miR-181a overexpression significantly blunted this response (Figures 7A, B). These data indicate that miR-181a confers protection against oxidative stress in cardiomyocytes, further supporting its role in limiting maladaptive cellular stress responses associated with heart failure pathology.

### 3.7 miR-181a enhances *β*-adrenergic-stimulated cAMP production in cardiomyocytes

**Figure 8:**
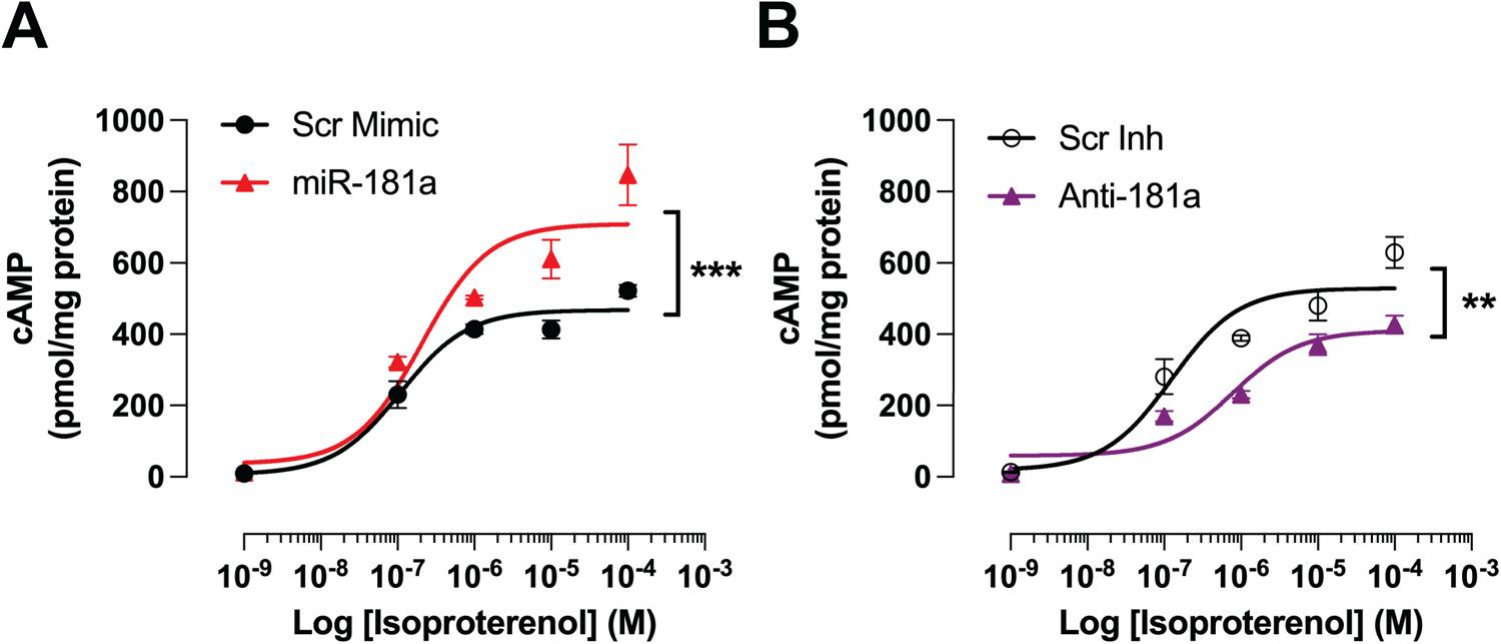
miR-181a regulates *β*-adrenergic-stimulated cAMP signaling in NRVMs. Intracellular cAMP levels were measured in NRVMs transfected for 48 h with miRNA mimics or inhibitors and stimulated with increasing concentrations of isoproterenol (ISO; 0, 10^−^⁷, 10^−^⁶, 10^−^⁵, 10^−^⁴ M) for 5 min following IBMX pretreatment (1 mM, 10 min). cAMP content was quantified by ELISA and normalized to total protein (pmol/mg). **(A)** miR-181a overexpression significantly increased ISO-stimulated cAMP accumulation compared with scrambled mimic controls, with statistical significance observed at the highest ISO concentration (10^−^⁴ M). Separation between curves became evident at intermediate ISO doses (≥10^−^⁶ M), consistent with enhanced *β*-adrenergic signaling capacity. **(B)** Inhibition of endogenous miR-181a significantly reduced ISO-stimulated cAMP accumulation compared with scrambled inhibitor controls at the highest ISO concentration (10^−^⁴ M), with divergence between groups apparent beginning at low ISO doses (≥10^−^⁷ M). Data are presented as individual biological replicates with mean ± SEM. *n* = 3 independent NRVM isolations, each measured in technical duplicate (duplicates averaged per biological replicate). Statistical analysis was performed using two-way ANOVA with Tukey's *post hoc* multiple comparisons test, where ** *p* < 0.01; *** *p* < 0.001.

Because GRK2-mediated *β*-AR desensitization directly impairs cAMP signaling (27, 28), we next investigated the impact of miR-181a on isoproterenol-induced cAMP production. NRVMs were transfected with either miR-181a mimic or anti-miR-181a for 48 h. Cells were pretreated with IBMX to prevent cAMP degradation, followed by exposure to increasing concentrations of isoproterenol (0, 10^−7^, 10^−6^, 10^−5^, 10^−4^ M). Scrambled mimic and scrambled inhibitor served as respective controls. Overexpression of miR-181a significantly enhanced intracellular cAMP accumulation across isoproterenol doses compared to the scrambled mimic control, indicating enhanced *β*-AR signaling (Figure 8A). In contrast, inhibition of endogenous miR-181a resulted in significantly blunted cAMP levels relative to the scrambled inhibitor control, demonstrating impaired *β*-AR-dependent cAMP generation (Figure 8B). Together, these findings support the notion that miR-181a enhances *β*-adrenergic signaling capacity, consistent with its ability to reduce GRK2 expression and limit receptor desensitization.

## 4 Discussion

In the present study, we conducted a miRNA microarray analysis of 2-week post-MI mouse hearts to investigate whether GRK2 is subject to miRNA regulation. The microarray yielded approximately 1,900 miRNAs, which were thoroughly examined for seed sequence complementarity to the GRK2 3′UTR. Further assessment of complementarity miRNA seed sequences using luciferase reporter assays identified a functional interaction between miR-181a and the GRK2 3′UTR.

Although miR-181a was not significantly downregulated post-MI in our microarray screen, several independent considerations supported its prioritization as a biologically meaningful GRK2 regulator. First, our selection strategy was centered on the capability of a miRNA to target GRK2 3′UTR rather than dysregulation of the miRNA itself post-injury. Thus, candidate selection was driven primarily by predicted targeting strength rather than absolute fold-change following MI. miR-181a demonstrated a high TargetScan context++ score and a conserved seed sequence, indicating a high probability for its ability to functionally repress GRK2. Second, miR-181a was expressed at significantly higher basal levels than other miR-181 family members in both sham and MI hearts, suggesting that it represents the predominant, physiologically relevant family member to exert regulatory control in the heart. Finally, prior reports implicating the cardioprotective role of miR-181a in ischemic injury (29, 30) further supported its functional plausibility in HF contexts. Together, these considerations provided strong mechanistic rationale to pursue miR-181a as a GRK2-targeting miRNA, even in the absence of large shifts in expression post-MI.

miR-181a has been previously shown to regulate multiple components of stress-response signaling pathways relevant to cardiovascular disease (29, 31, 32). Notably, miR-181a directly targets PTEN in cardiomyocytes, relieving inhibition of the PI3 K/AKT pathway and promoting cardioprotection during ischemia-reperfusion (I/R) injury (22). In the present study, we confirmed that miR-181a overexpression decreased PTEN mRNA levels, while miR-181a inhibition increased PTEN expression (Supplementary Figures S3A, B), validating functional miR-181a modulation in our experimental approach. Of note, PTEN analysis was included as an independent control experiment to validate the efficiency of miR-181a overexpression or inhibition rather than a central mechanistic focus, allowing us to interpret downstream signaling effects in the context of established miR-181a biology.

In NRVMs, miR-181a overexpression attenuated PE-induced GRK2 mRNA upregulation, whereas inhibition of endogenous miR-181a sustained or exacerbated GRK2 expression. Further, overexpression of miR-181a resulted in reduced cardiomyocyte surface area as well as expression levels of hypertrophic markers (NPPA/NPPB/MYH7) while improving cardiomyocyte survival following exposure to hypoxia. Finally, *β*-adrenergic-induced cAMP production was enhanced by miR-181a and diminished when miR-181a was inhibited. These findings collectively support a role for miR-181a as a post-transcriptional regulator of GRK2 that may mitigate pathological signaling and improve cellular resilience under stress.

GRK2 plays a central role in *β*-AR desensitization and maladaptive cardiac remodeling, and its upregulation is consistently reported in the failing human myocardium and in preclinical models of MI, hypertrophy, and chronic catecholaminergic stimulation (33, 34). Beyond canonical receptor desensitization, GRK2 influences Akt signaling, calcium handling, metabolic regulation, and mitochondrial function (7, 24, 35–38). Thus, identifying endogenous regulatory mechanisms that suppress GRK2 is biologically important and may have therapeutic relevance.

Our findings provide evidence that miR-181a contributes to the regulation of GRK2 expression in cardiomyocytes. The ability of miR-181a to suppress GRK2 in the setting of *β*-AR or *α*1-AR stimulation suggests miRNA-mediated feedback may exist to counterbalance stress-induced GRK2 accumulation. Conversely, miR-181a inhibition disrupts this control, resulting in persistent GRK2 elevation and sustained pathological signaling. These observations support a post-transcriptional component of GRK2 regulation and complement prior work demonstrating peptide or pharmacologic GRK2 targeting (8, 10, 39, 40).

While these findings support a cardioprotective role for miR-181a through GRK2 inhibition, prior studies have reported cell type-dependent and, in some cases, opposing effects of miR-181a in cardiovascular disease. Specifically, miR-181a overexpression has been shown to exacerbate pathology while miR-181a inhibition was reported to improve disease outcomes (41–43). Such discrepancies are likely due to the pleiotropic characteristic of miRNAs, which can regulate multiple targets in a context- and cell type-dependent manner (44, 45). In the present study, our data highlight GRK2 as a mechanistically relevant miR-181a target in cardiomyocytes. However, these findings also underscore the importance of interpreting miRNA function within specific biological contexts, particularly when considering therapeutic applications. The broad regulatory scope of miRNAs necessitates caution, as modulation of a single miRNA may have unintended effects across multiple signaling pathways.

The *β*-AR signaling pathway is essential for enhancing cardiac contractility and maintaining function under stress, however chronic stimulation drives pathological GRK2 elevation and *β*-AR dysfunction (46, 47). Our data suggest miR-181a contributes to maintaining *β*-AR responsiveness by preventing excessive GRK2 accumulation, thereby enhancing cAMP signaling following isoproterenol stimulation. This aligns with prior work implicating miR-181a in PI3 K/AKT pathway regulation by targeting PTEN (22), suggesting miR-181a may serve as a multifunctional regulator in stress signaling networks.

The reduction of NPPA, NPPB, and MYH7 expression levels with miR-181a overexpression, along with the exacerbated response following miR-181a inhibition, further support the a role for GRK2 repression in limiting hypertrophic signaling (34, 48). These findings place miR-181a upstream of key pathological processes, including *β*-AR dysfunction and hypertrophic remodeling.

Beyond its role in canonical *β*-AR signaling, GRK2 has been implicated in mitochondrial dysfunction, bioenergetic impairment, and cell death following cardiac injury (37, 38). The reduction in oxidative stress observed with miR-181a overexpression, together with improved cardiomyocyte survival under hypoxia, is consistent with these reports. Although our study does not directly examine mitochondrial GRK2 signaling, these findings align with the aforementioned reports in which inhibition of GRK2 prevented energetic collapse and promoted survival under ischemic conditions. In parallel, attenuation of hypertrophic gene expression suggests miR-181a simultaneously limits structural remodeling. These results support a mechanism of cardioprotection encompassing *β*-AR signaling preservation, suppression of pathological hypertrophy, and enhanced cell survival.

Several limitations of our study should be acknowledged. First, GRK2 targeting was not confirmed with a rescue strategy; thus, although consistent with GRK2 regulation, we cannot exclude miR-181a effects mediated through additional established targets. Second, our study is limited to *in vitro* models. While these systems provide mechanistic insight, they do not fully recapitulate the complex physiological condition of HF. Finally, although AKT signaling trends were explored, a direct mechanistic link between miR-181a, GRK2 suppression, and AKT signaling remains to be fully established.

Future work will extend these findings into translationally relevant *in vivo* models of HF. Specifically, adeno-associated virus serotype-9 (AAV9)-mediated delivery of miR-181a in murine models of MI and transverse aortic constriction (TAC) will determine whether miR-181a can modulate disease progression and improve cardiac function. Mechanistic studies using GRK2 rescue constructs lacking the 3′UTR will further clarify the specificity of miR-181a-mediated effects. Finally, comprehensive pathway analyses will be required to fully define how miR-181a regulates interconnected signaling networks in HF.

## 5 Conclusion

In this study, we identified miR-181a as a functional post-transcriptional regulator of GRK2 in cardiomyocytes and demonstrated that enhancing miR-181a expression limits pathological GRK2 upregulation in response to neurohumoral stress involving *β*-AR and *α*1-AR signaling. miR-181a overexpression attenuated hypertrophic signaling, preserved *β*-adrenergic responsiveness, and enhanced cell survival under hypoxic conditions, whereas inhibition of endogenous miR-181a produced opposing effects. These findings support a model in which miR-181a acts as an intrinsic cardioprotective modulator that stabilizes *β*-adrenergic signaling and mitigates maladaptive remodeling through suppression of GRK2. Our results provide mechanistic insight into miRNA-mediated regulation of GRK2 and highlight miR-181a as a potential therapeutic candidate for limiting pathological signaling and cellular injury in HF.

## Data Availability

The data discussed in this publication have been deposited in NCBI's Gene Expression Omnibus (49) and are accessible through GEO Series accession number GSE317550 (https://www.ncbi.nlm.nih.gov/geo/query/acc.cgi?acc=GSE317550).
