# Supplementary material for: miR-181a post-transcriptionally targets GRK2 to limit maladaptive signaling in cardiomyocytes
Source: Front Cardiovasc Med. 2026 Apr 22;13:1821660. doi: 10.3389/fcvm.2026.1821660 (PMC13144137; doi:10.3389/fcvm.2026.1821660)
Supplement: Supplementary file 1 [file Datasheet1.docx]

Supplementary Material

# Supplementary Figures

#
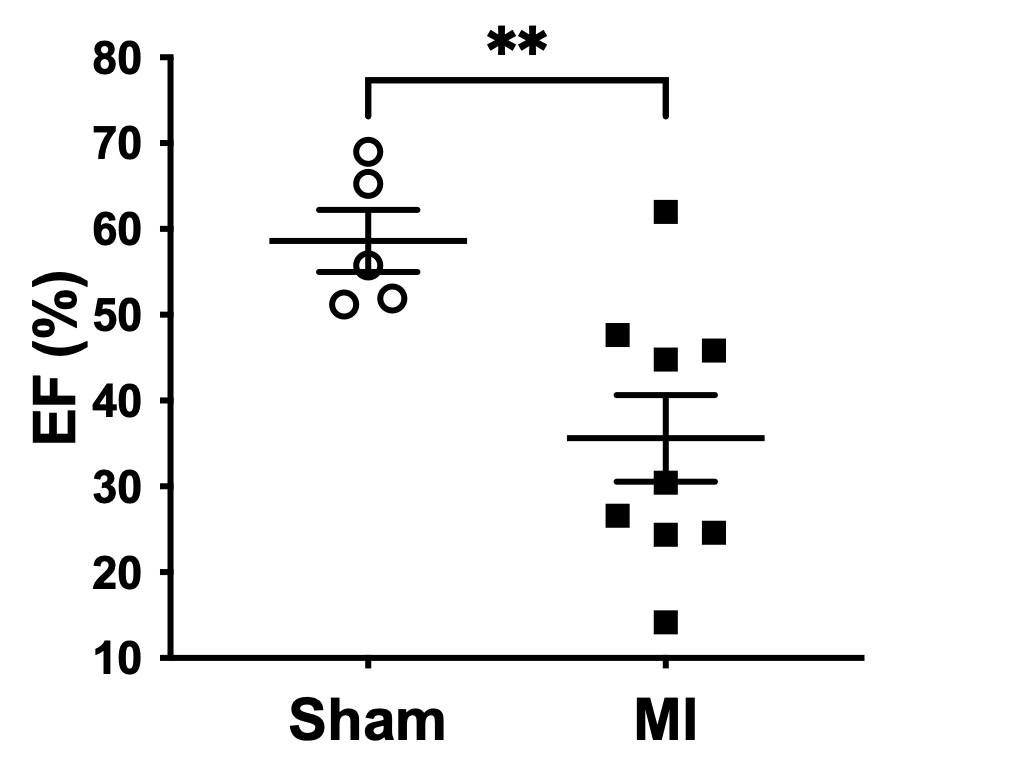


Figure S 1. Ejection fraction (EF, %) of 2-week post-MI mice. EF was measured by echocardiography in sham and 2-week post-MI mice (n=5, 9). Statistical significance was determined using unpaired two-tailed Student’s t-test comparing sham vs MI, where ** *p* < 0.01.


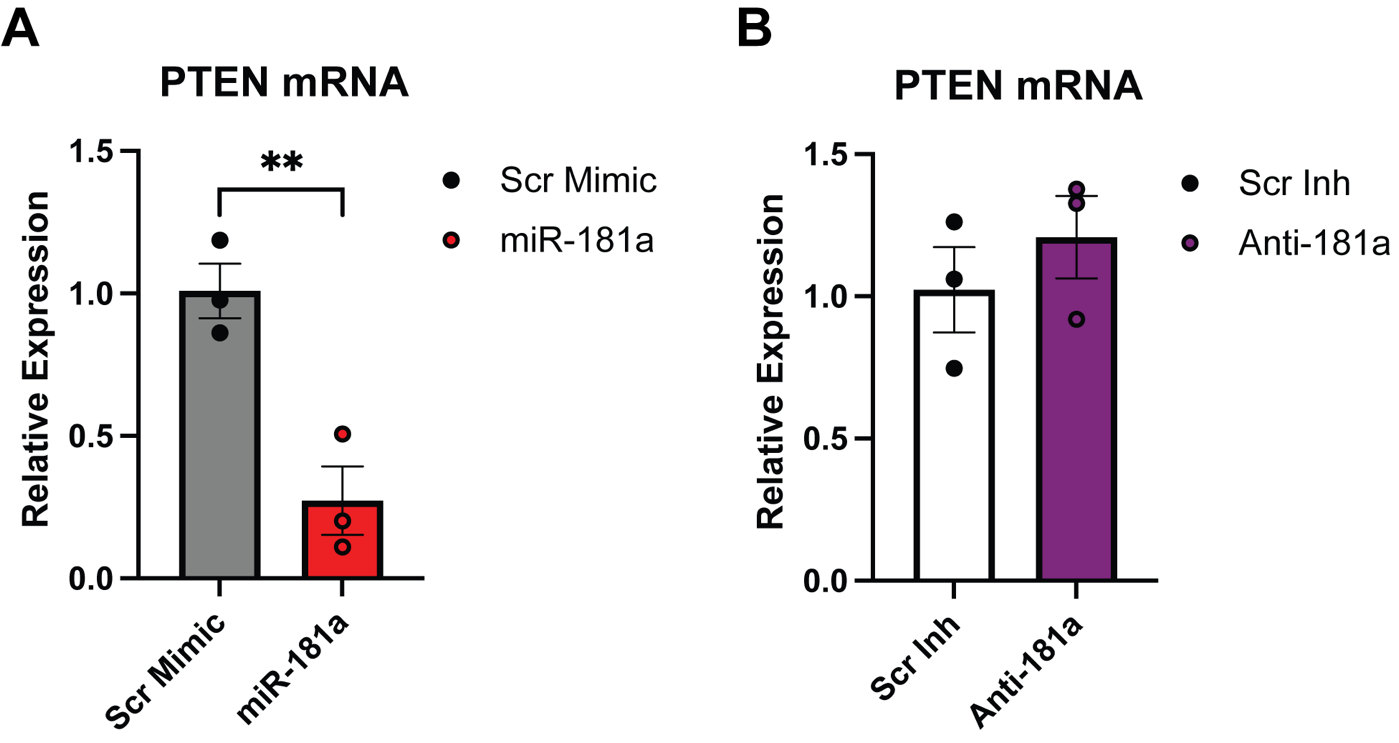


Figure S 2. miR-181a modulates expression of the established target PTEN in cardiomyocytes. (A) Quantitative RT-PCR analysis of PTEN mRNA expression in NRVMs transfected with miR-181a mimic or scrambled mimic control (scr mimic). miR-181a overexpression significantly reduced PTEN mRNA levels, consistent with published reports identifying PTEN as a direct miR-181a target. (B) Quantitative RT-PCR analysis of PTEN mRNA expression following transfection with anti-miR-181a (anti-181a) or scrambled inhibitor control (scr inh). Inhibition of miR-181a resulted in increased PTEN mRNA expression. Data are presented as individual data points with mean ± SEM, where n=3 independent NRVM isolations. Statistical significance was determined using unpaired two-tailed Student’s t-test, where * *p* < 0.05; ** *p* < 0.01.


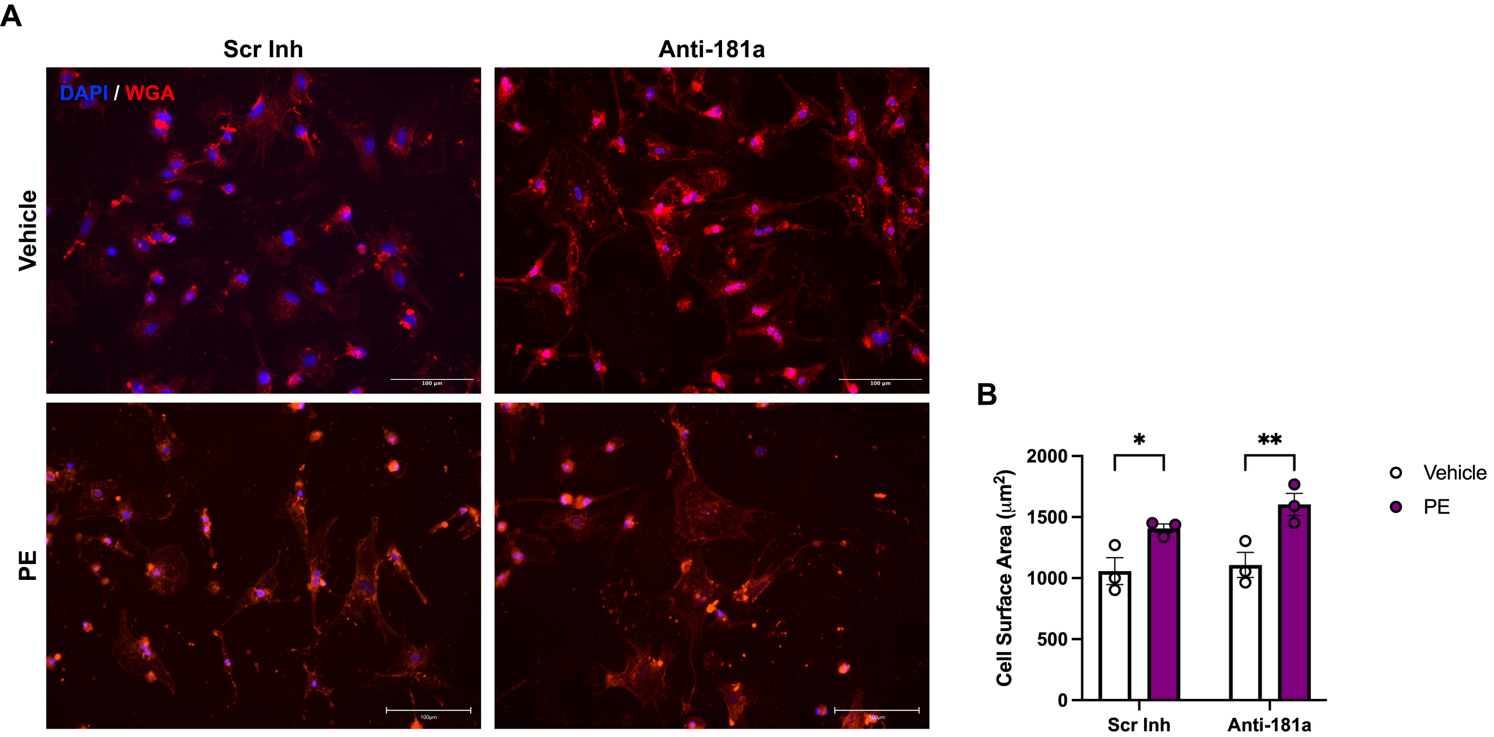


Figure S 3. Inhibition of miR-181a does not reduce PE-induced cardiomyocyte hypertrophy. NRVMs were transfected with anti-miR-181a (anti-181a) or scrambled inhibitor control for 24 hours and subsequently treated with 10 µM of PE for 48 hours to induce hypertrophic stress. Cardiomyocyte size was assessed by WGA staining. (A) Representative fluorescence images of NRVMs stained with Alexa Fluor 594-conjugated WGA following vehicle or PE treatment in scrambled inhibitor and anti-181a-transfected cells. (B) Quantification of cardiomyocyte surface area demonstrating that inhibition of miR-181a did not significantly reduce PE-induced increases in cell size compared with scrambled inhibitor controls. Data are presented as individual biological replicates with mean ± SEM. n = 3 independent NRVM isolations. For WGA analysis, two technical replicates per condition were analyzed per biological replicate, with six non-overlapping fields of view imaged per technical replicate; cell area measurements were averaged within technical replicates and then averaged per biological replicate. Statistical analysis was performed using two-way ANOVA with Tukey’s post-hoc multiple comparisons test, where * *p* < 0.05; ** *p* < 0.01.


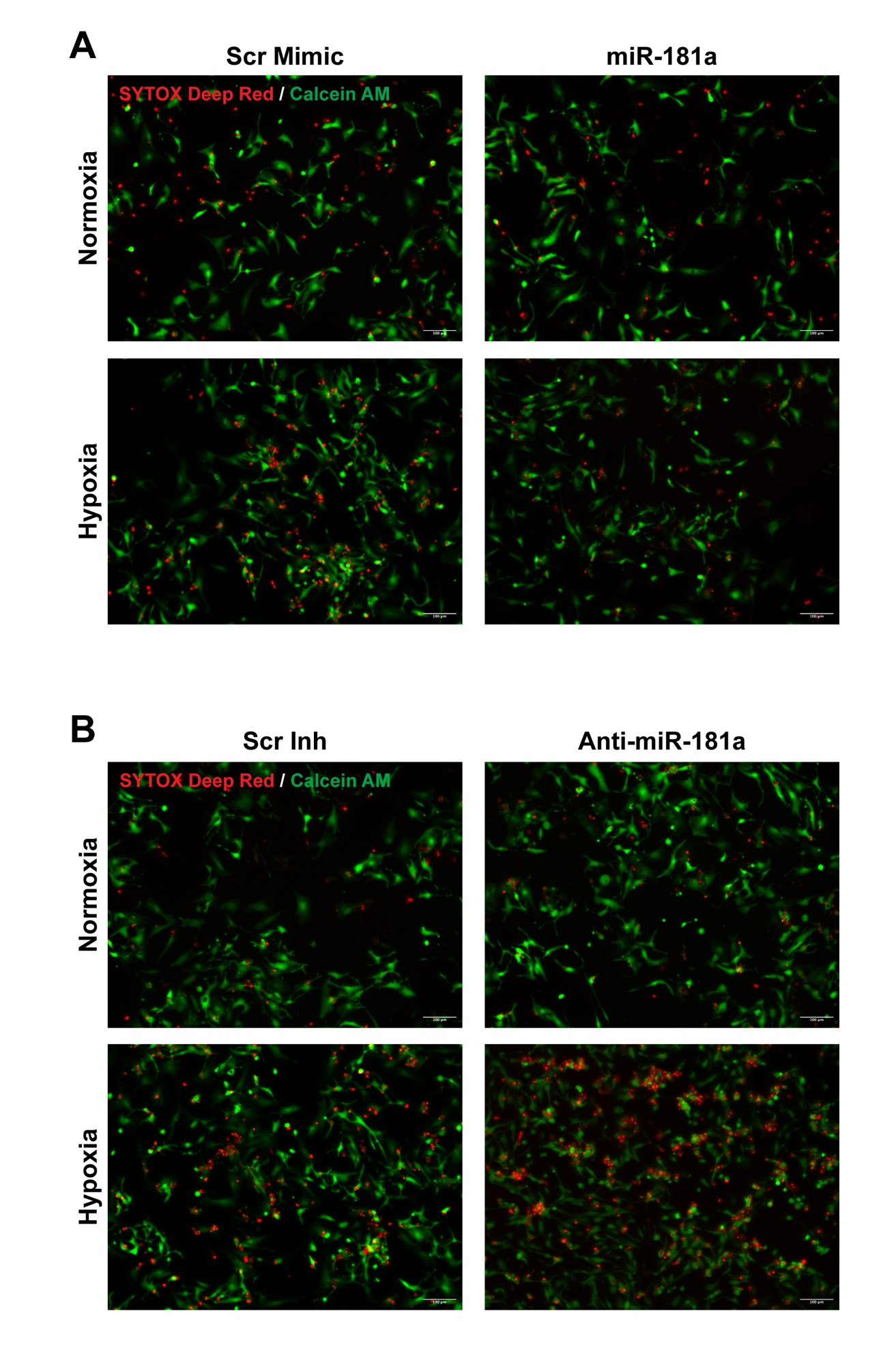


Figure S 4. Representative images of cardiomyocyte viability under hypoxic stress. NRVMs were subjected to normoxic or hypoxic conditions following modulation of miR-181a expression. (A) NRVMs transfected with scrambled mimic or miR-181a mimic. (B) NRVMs transfected with scrambled inhibitor or anti-miR-181a. Live cells were labeled with calcein AM (green; GFP channel), and dead cells were labeled with SYTOX Deep Red (red; CY5 channel). For each condition, six fields of view were acquired per well, with three technical replicate wells per group. Experiments were performed across three independent biological replicates (separate NRVM isolations). Scale bars, 100 µm.
